# Global transcriptional analysis of *Burkholderia pseudomallei* high and low biofilm producers reveals insights into biofilm production and virulence

**DOI:** 10.1186/s12864-015-1692-0

**Published:** 2015-06-20

**Authors:** Chui-Yoke Chin, Yuka Hara, Ahmad-Kamal Ghazali, Soon-Joo Yap, Cin Kong, Yee-Chin Wong, Naufal Rozali, Seng-Fook Koh, Chee-Choong Hoh, Savithri D. Puthucheary, Sheila Nathan

**Affiliations:** School of Biosciences and Biotechnology, Faculty of Science and Technology, Universiti Kebangsaan Malaysia, 43600 Bangi, Selangor Darul Ehsan Malaysia; Codon Genomics SB, Seri Kembangan, Selangor D.E. Malaysia; Malaysia Genome Institute, Jalan Bangi, Kajang, Selangor D.E. Malaysia; Department of Medical Microbiology, Faculty of Medicine, University of Malaya, Kuala Lumpur, Malaysia; Present address: Emory Vaccine Centre, Emory University, Atlanta, Georgia USA; Present address: Centre for Traditional Chinese Medicine, INTI International University, Nilai, Selangor Malaysia; Present address: Medical Education, Research and Evaluation Department, Duke-NUS Graduate Medical School Singapore, Singapore, Singapore

**Keywords:** *B. pseudomallei*, Biofilm, Transcriptome

## Abstract

**Background:**

Chronic bacterial infections occur as a result of the infecting pathogen’s ability to live within a biofilm, hence escaping the detrimental effects of antibiotics and the immune defense system. *Burkholderia pseudomallei*, a gram-negative facultative pathogen, is distinctive in its ability to survive within phagocytic and non-phagocytic cells, to persist *in vivo* for many years and subsequently leading to relapse as well as the development of chronic disease. The capacity to persist has been attributed to the pathogen’s ability to form biofilm. However, the underlying biology of *B. pseudomallei* biofilm development remains unresolved.

**Results:**

We utilised RNA-Sequencing to identify genes that contribute to *B. pseudomallei* biofilm phenotype. Transcriptome analysis of a high and low biofilm producer identified 563 differentially regulated genes, implying that expression of ~9.5 % of the total *B. pseudomallei* gene content was altered during biofilm formation. Genes involved in surface-associated motility, surface composition and cell wall biogenesis were over-expressed and probably play a role in the initial attachment of biofilms. Up-regulation of genes related to two component signal transduction systems and a denitrification enzyme pathway suggest that the *B. pseudomallei* high biofilm producer is able to sense the surrounding environmental conditions and regulate the production of extracellular polymeric substance matrix, a hallmark of microbial biofilm formation.

**Conclusions:**

The transcriptome profile described here provides the first comprehensive view of genes that contribute to the biofilm phenotype in *B. pseudomallei*.

**Electronic supplementary material:**

The online version of this article (doi:10.1186/s12864-015-1692-0) contains supplementary material, which is available to authorized users.

## Background

Bacterial cells have evolved a biofilm phenotype over billions of years as part of their successful strategy to colonize biotic and abiotic surfaces when faced with different environmental conditions. Biofilm consists of a biological architecture of aggregated microbes on a surface, enclosed within a mesh of exopolysaccharides, fatty acids, DNA and large surface proteins [1]. Biofilms are closely associated with persistence as the presence of the extracellular matrix surrounding the cells renders the bacteria less susceptible to anti-bacterial agents compared to free-floating cells [2]. As a result, biofilm infections tend to be chronic and are difficult to eradicate. The transition from free-swimming planktonic cells to biofilm producers occurs in response to environmental changes including pH, temperature, nutrient levels and ionic strength. This response involves multiple regulatory networks which translate signals to alter the reorganization of the bacterial cell to survive unfavourable conditions [3]. It is generally believed that quorum sensing contributes to the formation of a functioning biofilm. Human infections involving biofilm have been described in patients with native valve endocarditis, cystic fibrosis, periodontitis as well as chronic bacterial infections such as prostatitis. Biofilm formation in medical devices such as central venous and urinary catheters, prosthetic heart valves, intrauterine devices and contact lenses, is well described [4].

*Burkholderia pseudomallei*, the causative agent of melioidosis, is known to produce biofilm. A major feature of melioidosis is the difficulty in achieving complete bacterial eradication following an episode of infection and an extended period of antimicrobial treatment is needed for total clearance. Formation of biofilm has been proposed as a contributory factor in the occurrence of persistent infection in the host. Clinical response to antimicrobials is slow and recurrent disease is common [5]. Sawasdidoln et al. [6] demonstrated that *B. pseudomallei* isolates which were sensitive to doxycycline, ceftazidime, imipenem and trimethoprim/sulfamethoxazole became resistant under conditions that promoted the formation of biofilm.

Levels of humoral antibodies in patients who have had melioidosis remain high and seldom drop to basal level even years after recovery from an acute infection, supporting the notion of persistence [7]. It is possible that *B. pseudomallei* can adapt to survival *in vivo* through the formation of biofilm but the mechanism by which this occurs in melioidosis patients is unclear [8]. It has also been reported that *B. pseudomallei* biofilm does not contribute to the virulence of the organism [9]. Based on studies involving various *B. pseudomallei* mutants, acapsular mutants may or may not have reduced formation of biofilm [6, 10]. On the other hand, restricted biofilm formation was observed in the *fliC* flagella mutant [6] and the polyphosphate kinase *ppk* mutant [11] whilst the role of *B. pseudomallei* cyclic-di-GMP-phosphodiesterase (CdpA) in biofilm formation and virulence was established with the corresponding *cdpA* mutant being attenuated in human macrophage cells [12]. A recent report by Lazar-Adler et al. [13] proposed the role of *B. pseudomallei* Trimeric Autotransporter Adhesins (TAA) in biofilm formation whereby an insertional mutant of the *BPSS1439* gene was affected in its ability to form biofilm in addition to being partially attenuated in an acute murine melioidosis model, implying a positive relationship between biofilm formation and bacterial virulence.

A number of studies involving individual mutants of the biofilm-associated genes described above have demonstrated that inactivating these single genes does not completely attenuate biofilm formation. This suggests a more global regulation of multiple *B. pseudomallei* genes and pathways involved in biofilm formation and may, either directly or indirectly, be related to virulence or persistence in infected hosts. Hence, in this study, a comprehensive transcriptional analysis of representative high and low clinical *B. pseudomallei* biofilm producers was performed to identify the genes required for biofilm formation in *B. pseudomallei*. In addition, preliminary virulence studies of these two *B. pseudomallei* biofilm producers were carried out using the nematode *Caenorhabditis elegans* and BALB/c mice infection models.

## Results

### Transcriptome analysis and global transcriptional profile of *B. pseudomallei* biofilm strains

**Fig. 1:**
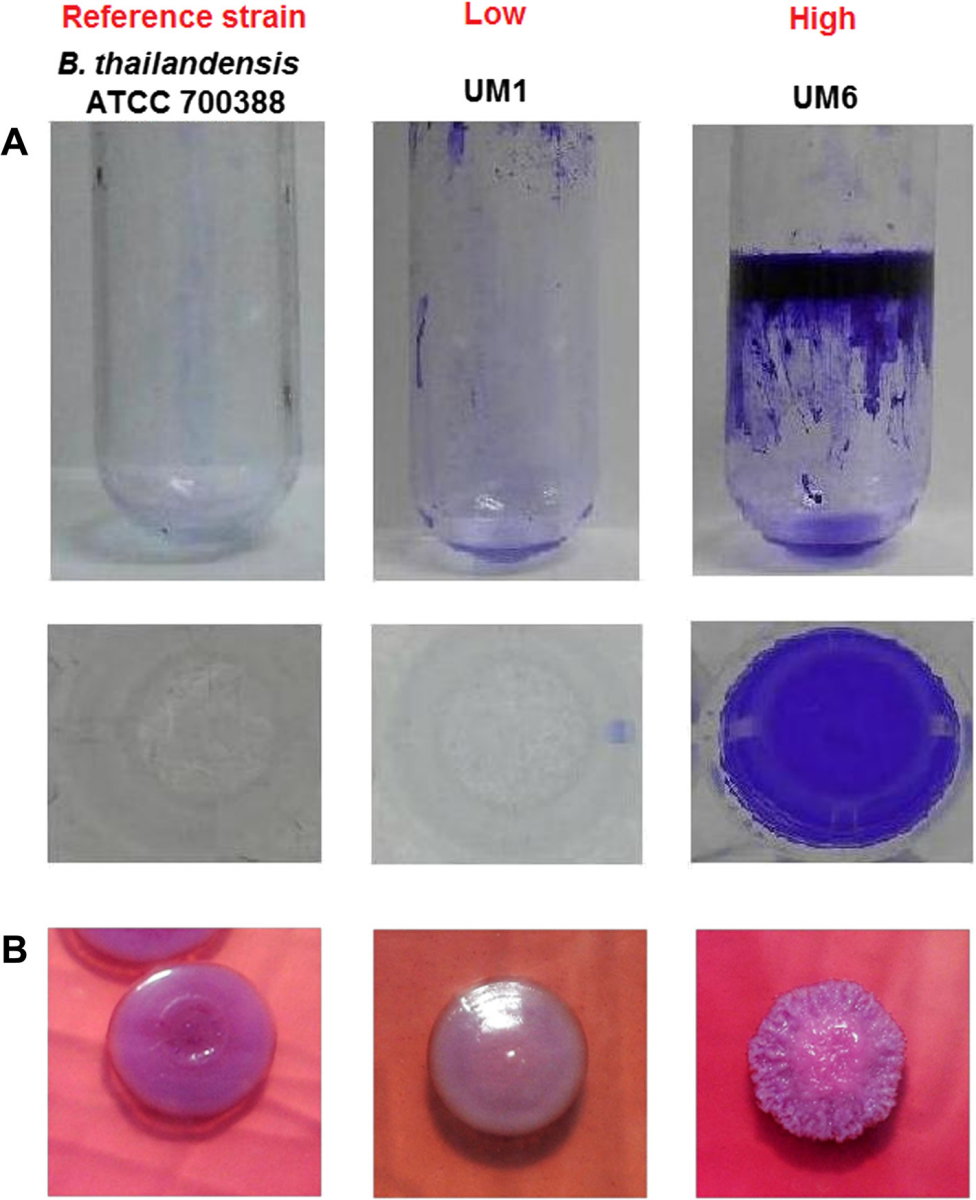
*B. pseudomallei* biofilm formation phenotypes. **a** Biofilm formation was measured by crystal violet staining assay using static broth cultures of *B. pseudomallei* UM1 (low producer) and UM6 (high producer) in a 96-well flat-bottomed microtiter plate and in test tubes. Non-pathogenic *B. thailandensis* ATCC 700388 was used as the reference strain in this study. **b** Colony morphology of *B. thailandensis* ATCC 700388, *B. pseudomallei* UM1 (low producer) and UM6 (high producer) on Ashdown agar plates after 48 h incubation at 37 °C

The sequence based transcriptome approach has been used to study regulatory mechanisms and pathogenicity factors of *Pseudomonas syringae* [14], *Mycobacterium tuberculosis* [15], *Pseudomonas aeruginosa* [16], *Listeria monocytogenes* and *Listeria innocua* [17]. We utilised RNA sequencing and comparative transcriptome analysis to identify genes and their respective expression levels that contribute to the *B. pseudomallei* biofilm phenotype. A total of 84 *B. pseudomallei* clinical isolates were analysed for biofilm formation (Additional file 1). From this collection, we selected one representative from the high biofilm producers, UM6, and one of the low biofilm producers, UM1 for RNA-Seq analysis. The biofilm formation phenotypes of both these strains is presented in Fig. 1. Both strains were sequenced on the Illumina platform and sequence reads were mapped to the annotated *B. pseudomallei* strain K96243 genome. The expression analysis demonstrated that approximately 84.5 % of the UM1 and UM6 reads mapping to *B. pseudomallei* K96243 genes had a calculable ‘fragments per kilobase of million fragments mapped’ (FPKM) value (Additional file 2). The pattern of relative gene expression was similar between the biological replicates with a correlation coefficient of r = 0.86 and r = 0.87 for UM6 and UM1, respectively.

**Fig. 2:**
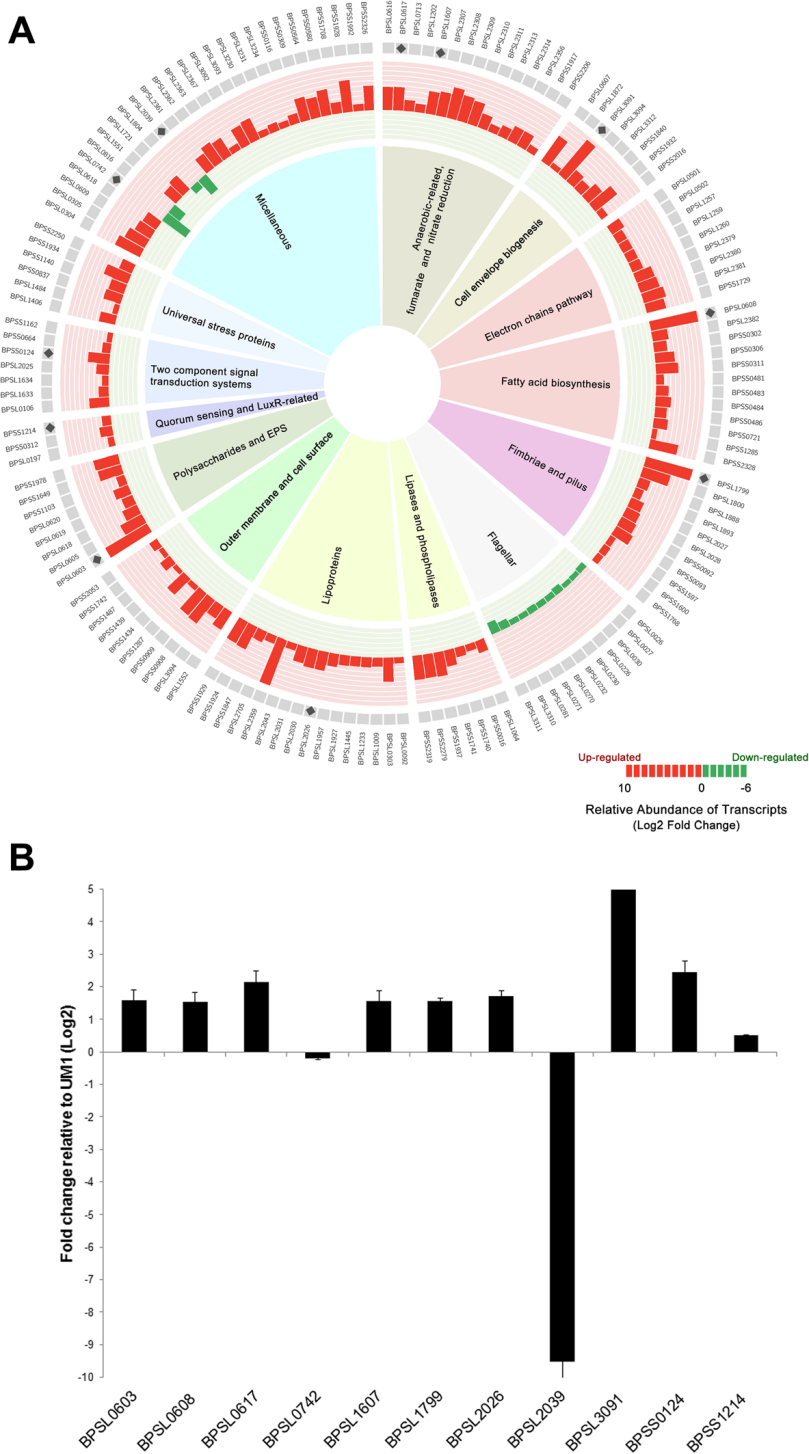
*B. pseudomallei* biofilm development transcriptome profile. **a** Expression profiles of modulated-genes according to functional categories. Transcript expression of log_2_ fold level are depicted by the histogram. The height of the bars correspond to the degree of expression level. Red and green bars represent up-and down-regulation in relative expression levels, respectively. **b** qRT-PCR analysis of eleven *B. pseudomallei* genes from seven functional categories differentially expressed as determined by RNA-Seq. The results are from a representative of three reproducible independent experiments

We next used the transcriptome data to identify genes that potentially contribute towards the biofilm phenotype in *B. pseudomallei* as determined by differential transcription analysis between UM6 and UM1. By adopting a q-value of ≤ 0.05 and log_2_ fold-change above 1 to classify a transcript as being differentially expressed, transcriptional analysis revealed 563 differentially expressed genes (324 up-regulated genes and 239 down-regulated genes) in UM6 relative to UM1. Functional classification of up- and down-regulated genes showed that most of these genes encode core functions such as cell envelope, central intermediary metabolism, energy metabolism, transport, regulatory proteins and cellular processes (Additional file 3). Many genes encoding proteins with unknown function or hypothetical proteins were also modulated in the high biofilm producer, UM6 (Additional file 3). Furthermore, genes predicted to encode proteins that are known to localise as extracellular proteins were observed at a higher percentage in the group of genes with up-regulated expression (Additional file 3). Eleven genes were randomly selected from seven functional categories (Fig. 2a and Additional file 4) for validation by quantitative real-time PCR (qRT-PCR). The expression was verified by qRT-PCR as up- or down-regulated, albeit with magnitudes different from those recorded by RNA-Seq (Fig. 2b and Additional file 5). In lieu of the large number of significantly differentiated genes, only data related to genes that have some functional information are shown and discussed below.

### Fimbriae and pilus may be required for initiation of *B. pseudomallei* biofilm attachment

**Fig. 3:**
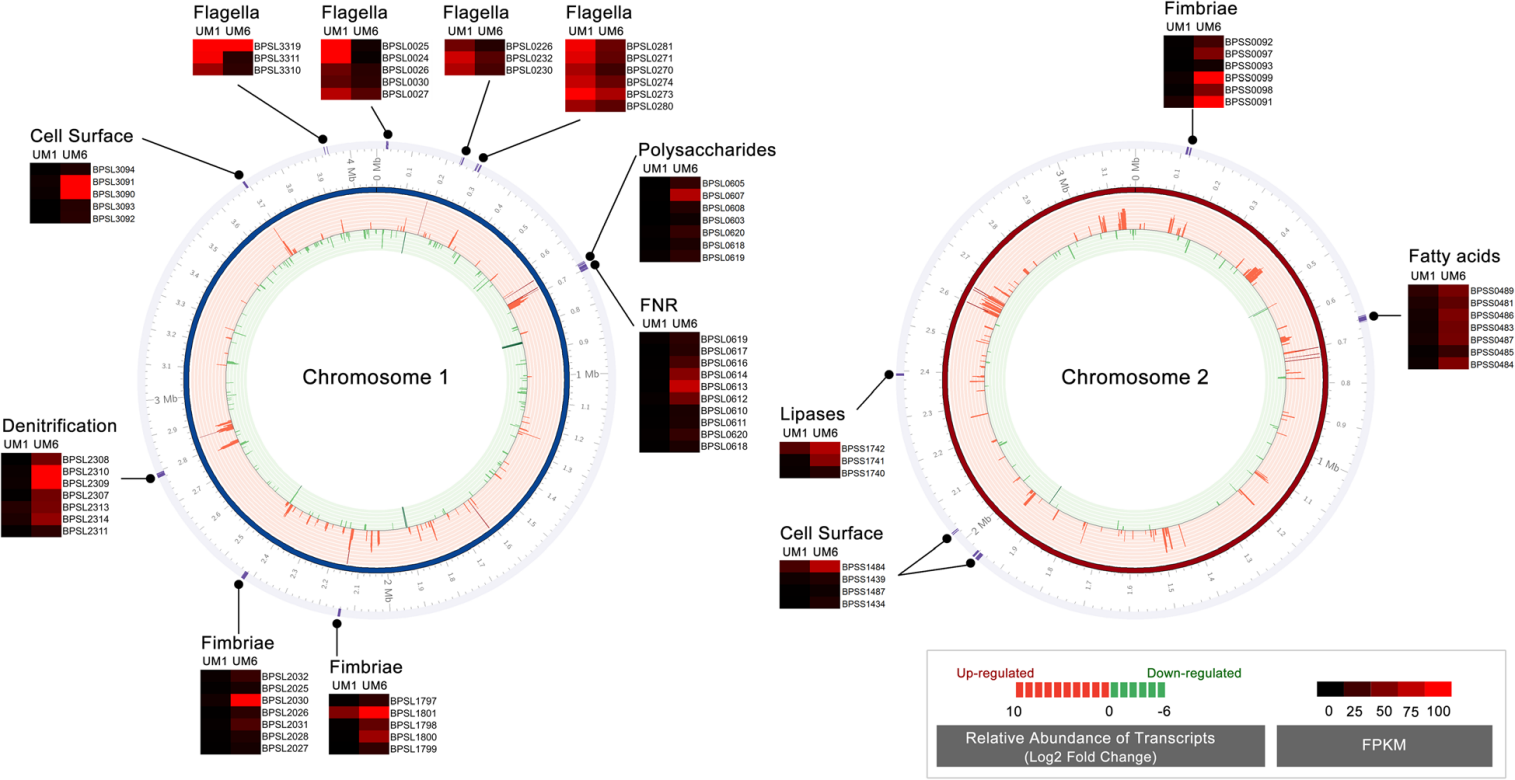
Alteration of surface composition and regulation of anaerobic respiration contribute to *B. pseudomallei* biofilm phenotype. Transcript expression of log_2_ fold level are depicted by the histogram in the inner-most ring of the circular maps. The height of the bars correspond to the degree of expression level. Genes uniquely expressed in UM6 and UM1 are coloured in dark red and dark green, respectively. Red and green bars represent up- and down-regulation in relative expression levels, respectively. Hierarchical clustering of *B. pseudomallei* UM1 (low biofim producer) and UM6 (high biofilm producer) expression profiles according to functional categories. The heat maps indicate the gene transcripts expressed as FPKM. Genes whose expression did not change are coloured in black

**Fig. 4:**
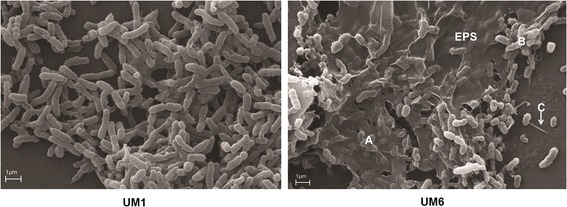
Scanning electron microscope micrograph of biofilm formation by *B. pseudomallei* on a glass slide. *B. pseudomallei* UM1demonstrated reduced biofilm formation compared to UM6. **a** Extracellular polymeric substance (EPS) is clearly visible around the *B. pseudomallei* UM6 colonies. **b** The matrix is holding the bacteria together but has not yet been encapsulated. **c** Pilus protruding from a UM6 colony

Motility influences biofilm formation in various pathogens including Enteropathogenic *E. coli* [18, 19] and *P. aeruginosa* [20, 21]. A number of fimbriae and pilus-related genes (*BPSL1799, BPSL1888, BPSL1893, BPSL2027, BPSL2028, BPSS0092, BPSS1597, BPSS1600* and *BPSS1768*) were significantly up-regulated in UM6 (Figs. 2a and 3 and Additional file 4) suggesting that these structures may also be important in *B. pseudomallei* biofilm attachment. To validate the observation of over-expressed pili-related genes, we performed scanning electron microscopy (SEM) on both strains. The micrographs (Fig. 4) demonstrate the presence of pili protruding from UM6 which are not observed in UM1, thus supporting the transcriptional-level analysis (Fig. 4).

To gain more insight into the identified genes, we used the Conserved Domain Database (CDD) to look at protein domains of these genes. *BPSL2027* (putative fimbriae-related protein) contains an usher superfamily domain (pfam0057) and PapC C-terminal (pfam13953) and N-terminal conserved domains (pfam13954). Concomitantly, two outer membrane usher proteins, *BPSS0093* (4.07 fold) and *BPSL1800* (5.99 fold), were over expressed in UM6 and may be involved in assembly of pre-pilins for fimbriae biogenesis. In contrast, a cohort of flagella-related genes encoding the flagella basal body, motor switch and hook proteins (*BPSL0026, BPSL0027, BPSL0030, BPSL0226, BPSL0230, BPSL0232, BPSL0270, BPSL0271, BPSL0281, BPSL3310* and *BPSL3311*) were down-regulated in UM6 (Figs. 2a and 3 and Additional file 4), similar to that previously described for *Pseudomonas aeruginosa* [22] and *Escherichia coli* biofilm formation [22]. The down-regulation of flagella-related genes was validated with motility assays performed on solid agar, which demonstrated that the UM6 strain has reduced swimming and swarming motility compared to the UM1 strain (Table 1).

**Table 1:** Swimming and swarming analysis of UM1 and UM6. Data are mean ± SD of two independent experiments

Phenotype	UM1	UM6
Swimming (mm in diameter)	69.55 ± 7.14*	53.4 ± 1.98
Swarming (mm in diameter)	74.55 ± 3.62*	55.82 ± 2.36

*p < 0.05 (Students’ *t* test)

### Over expression of extracellular polymeric substance (EPS) matrix components for development of *B. pseudomallei* biofilm architecture

Genes encoding for polysaccharides (*BPSL0603, BPSL0605, BPSL0618, BPSL0619, BPSL0620, BPSS1649*) and EPS (*BPSS1978*) were over-expressed in UM6 (Fig. 2a and Additional file 4). Observations based on SEM micrographs support the expression profile whereby the presence of EPS matrix encapsulating the bacteria was only seen in the high biofilm producer strain UM6 but not in the low biofilm producer strain UM1 (Fig. 4). Attachment of Gram-negative bacteria to a surface via outer membrane proteins is the first step in biofilm formation, followed by replication to form micro colonies and production of a mature biofilm [23]. Several outer membrane and cell-surface encoded proteins were also over-expressed in UM6, including *BPSS0908, BPSS0909, BPSS1287, BPSS1487, BPSS1742, BPSS1434, BPSS1439, BPSS2053, BPSL1552* and *BPSL3094* (Figs. 2 and 3 and Additional file 4).

### Alteration of *B. pseudomallei* surface composition in a high biofilm producer

Expression levels of 13 fatty acid biosynthesis-related genes (*BPSL0608, BPSL0618, BPSL2382, BPSS0302, BPSS0306, BPSS0311, BPSS0481, BPSS0483, BPSS0484, BPSS0486, BPSS0712, BPSS1285* and *BPSS2328*), seven phospholipases and lipase-related genes (*BPSL1064, BPSS0016, BPSS1740, BPSS1741, BPSS1937, BPSS2279* and *BPSS2319*) as well as seven cell envelope biogenesis-related genes (*BPSL0607, BPSL1872, BPSL3094, BPSL3312, BPSS1840, BPSS1932* and *BPSS2016*) were up-regulated in the high biofilm producer (Figs. 2 and 3 and Additional file 4). Furthermore, 13 lipoprotein-encoding genes (*BPSL0092, BPSL0303, BPSL1233, BPSL1445, BPSL1927, BPSL1957, BPSL2026, BPSL2043, BPSL2359, BPSL2705, BPSS1847, BPSS1924* and *BPSS1929*) were also over-expressed in UM6. One of the putative lipoproteins (*BPSL2026*) contained a spore coat protein U domain, SCPU (cl02253), which is generally present in the bacterial family of secreted pili proteins involved in motility and biofilm formation. Concomitantly, three putative SCPU domain containing export protein genes, *BPSL1009* (1.95 fold), *BPSL2030* (4.01 fold) and *BPSL2031* (3.51 fold), were also identified as over-expressed in UM6 (Fig. 3 and Additional file 4).

### LuxR-like domain is likely to be involved in *B. pseudomallei* biofilm formation

Quorum sensing (QS) is a form of cell to cell communication that bacteria adopt to coordinate group behaviour in a cell density dependent manner. QS relies on *N*-acyl homoserine lactones (AHLs) to regulate gene expression in response to cell density dependent cues and is related to biofilm formation and exopolysaccharide production [1, 20, 24]. In addition, QS influences the expression profile of diverse genes including antibiotic tolerance and virulence determinants [2]. The QS system plays a major role in the control of bacterial biofilm formation in many known pathogens including *P. aeruginosa* [20, 25], *Streptococcus pneumonia*e [26] and *E. coli* [27, 28]. In this study, the expression levels of homoserine O-acetyltransferase (*BPSL0197*) and the LuxR-family transcriptional regulator (*BPSS0312*), which, together mediate gene expression following association with the cognate AHL (s), were up-regulated in UM6 (Fig. 2a and Additional file 4).

### Up-regulation of two component signal transduction systems and stress proteins in the *B. pseudomallei* high biofilm producer

The two component signal (TCS) transduction system related proteins, a sensor histidine kinase protein and response regulator, are responsible in regulating biofilm formation in a number of bacteria. Several genes related to the two-component signal transduction systems (*BPSL0106, BPSL1633, BPSL1634, BPSL2025, BPSL2314, BPSS0124, BPSS0664* and *BPSS1162*) were up-regulated in UM6 (Fig. 2a and Additional file 4). Interestingly, two putative sensor kinases (*BPSL2025* and *BPSL1634*) demonstrated considerable similarity to the *E. coli* RcsC sensor protein, particularly at the conserved domains (Additional file 6).

Four genes encoding response regulators (*BPSL1633, BPSL2314, BPSS0124* and *BPSS1214*) that contained a LuxR-like domain (cd06170) were identified as up-regulated in UM6 (Fig. 2a and Additional file 4). Amongst the identified genes, a hypothetical protein (*BPSL0106*) containing the CpxP component domain (cl01482), was up-regulated by 4.1 fold. Proteins containing the Cpx component domain are known modulators of cell-envelope stress in Gram-negative bacteria including *E. coli* biofilm-producing cells [29]. In addition, genes encoding two universal stress proteins (*BPSS1140, BPSS1934*) and one hypothetical protein (*BPSS0837*) with a universal stress protein family domain (cd00293) as well as genes of three stress–related proteins (*BPSS2250, BPSL1484* and *BPSL1406*) were also up-regulated in UM6.

### Modulation of the denitrification enzyme pathway in the *B. pseudomallei* high biofilm producer

Two anaerobic-related genes (*BPSL2309* and *BPSL2356*), three reductase genes involved in nitrate metabolism (*BPSL2351*, *BPSL1607*, *BPSS1487*) and several genes encoding fumarate and the nitrate reduction (FNR) subfamily were over expressed in UM6 (Figs. 2a and 3 and Additional file 4). The majority of these genes encode for proteins involved in nitrate regulation and dissimilation including nitrate reductases (*BPSL2309, BPSL2310, BPSL2311*), nitrate-oxide reductase (*BPSL1607*), nitrate sensor protein (*BPSL2313*) and nitrate extrusion proteins (*BPSL2307, BPSL2308*) (Fig. 3 and Additional file 4). Of interest, two nitrite extrusion proteins and a transport-related membrane protein (*BPSS2206*) contain the major facilitator superfamily (MFS) domain (cd06174), which is involved in the symport, antiport or uniport pumping of various substrates such as sugars, oligosaccharides and antibiotics [30]. Moreover, one of the crp-family transcriptional regulators (*BPSS1917*) contained the effector domain of the CAP family transcription factor (cd00038) whilst two hypothetical proteins (*BPSL0616* and *BPSL0617*) that also contained the same domain, were up regulated (4.72 fold and 4.74 fold, respectively) in UM6 compared to UM1. Up-regulation of MFS-containing genes has recently been associated with the development of biofilm by *P. aeruginosa* [31] as well as adherence and biofilm formation for *Acinetobacter baumannii* [30].

### Potential correlation between *B. pseudomallei* biofilm formation and virulence in nematode and mice models

**Fig. 5:**
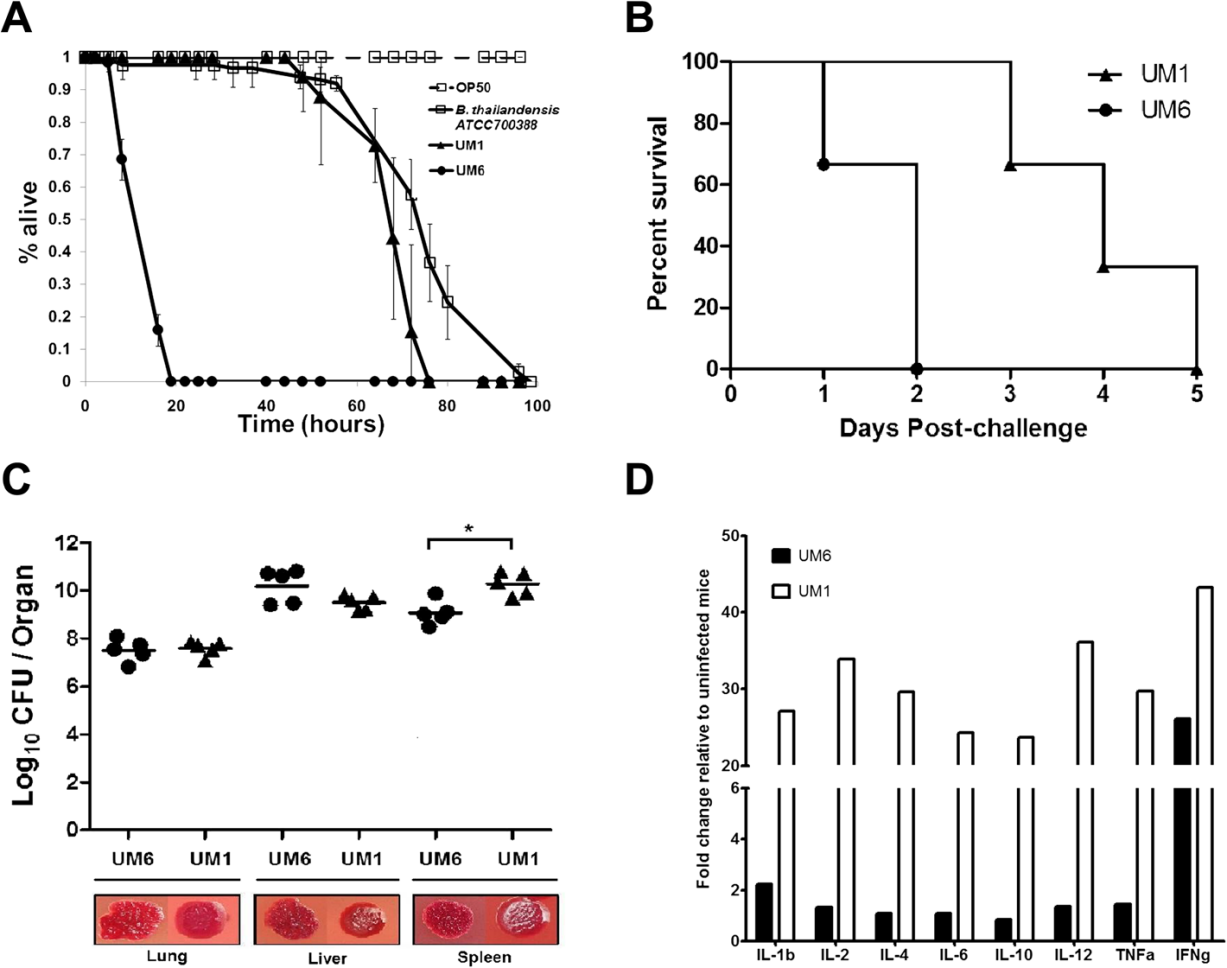
*B. pseudomallei* biofilm may contribute to bacterial pathogenesis. **a** *B. pseudomallei* biofilm contributes towards lethality in *C. elegans*. One-day old Glp worms were transferred to individual *B. pseudomallei* isolates UM1 (black line, solid triangles), UM6 (black line, solid circles), *B. thailandensis* ATCC 700388 (black line, open squares) and *E. coli* OP50 (black dashed line, open squares). The graph shows the mean ± SD of three replicates (30 worms/replicate) from a representative of two independent experiments. **b** Mice (n = 5) were challenged intraperitonealy with a lethal dose of *B. pseudomallei* UM6 (circle) or UM1 (triangle) and their survival was monitored. Mice challenged with UM6 succumbed to disease significantly faster (within 24 h) than those challenged with UM1 [Logrank (Mantel-Cox) test, *p*-value = 0.0084]. **c** The bacterial loads in the spleen, liver and lung of *B. pseudomallei*-infected mice are shown. Each symbol represents one mouse. The horizontal line indicates the geometry mean for each group. Significance was determined using the Mann–Whitney test (*p < 0.05). Representative colony morphologies of *B. pseudomallei* UM1 and UM6 harvested from the infected mice organs are shown. **d** *B. pseudomallei* UM6 attenuates production of various cytokines. Lungs of BALB/c mice challenged with *B. pseudomallei* UM1 or UM6 were harvested and the levels of 12 pro- and anti-inflammatory cytokines were measured by the mouse cytokine Multi-Analyte ELISArray Kit (Qiagen). Cytokine expression is shown as fold increase compared to the control unchallenged mice

Biofilm formation has been implicated as a virulence factor in *C. elegans* infection models for *Yersinia pseudotuberculosis* [32] and staphylococcal infections [33]. Hence, we used the *C. elegans* host model to evaluate virulence of the different *B. pseudomallei* biofilm producers and determine the contribution of biofilm in *B. pseudomallei*-mediated killing of *C. elegans.* Nematodes were fed with *B. pseudomallei* UM1 (low biofilm producer) and UM6 (high producer), respectively, and the non-pathogenic *B. thailandensis* ATCC 700388 [34]. As shown in Fig. 5a, worms exposed to the laboratory food source *E. coli* OP50 remained completely viable over the course of the experiment. Worms exposed to UM6 died significantly faster (Logrank (Mantel-Cox) test *p* < 0.0001) with a mean time to death (TD_mean_) of 13.897 ± 0.401 h compared to worms exposed to *B. thailandensis* (TD_mean_ = 77.631 ± 1.638 h) and UM1 (TD_mean_ = 77.876 ± 1.183 h). The preliminary survival/virulence assay demonstrates that *B. pseudomallei* biofilm production could be a contributing virulence factor in the pathogenesis of this bacterium.

BALB/c mice serve as a well-established animal model for melioidosis. To confirm the findings in *C. elegans*, mice were challenged intraperitoneally with a lethal dose of UM6 or UM1 and mice survival was monitored. As observed in *C. elegans*, mice infected with UM6 died significantly faster than those infected with UM1 (Fig. 5b). All mice infected with UM6 succumbed to disease within 24 h with a median survival of 1 day while only 1 mouse infected with UM1 died on day one and the remaining 4 mice succumbed to disease on day 3 with a median survival of 3 days. Although UM6 appeared to be more virulent, bacterial loads in the lungs and livers of UM6 and UM1-infected mice were similar, and the spleens of UM6 infected mice displayed a significantly lower bacterial count compared to UM1 infected mice (Fig. 5c).

The high biofilm producer was able to kill both mice and nematode relatively quickly suggesting an imbalance between the host proinflammatory and anti-inflammatory responses towards infection. Hence, we asked if the presence of the biofilm deregulated this equilibrium by limiting the cytokine response to infection. To address this question, the mouse cytokine Multi-Analyte ELISArray Kit (Qiagen) was utilised to simultaneously measure 12 cytokines i.e., IL-1α, IL-1β, IL-2, IL-4, IL-6, IL-10, IL-12, IL-17A, Interferon-γ (IFN-γ), Tumour necrosis factor-α (TNF-α), Granulocyte- Colony Stimulating Factor (G-CSF) and Granulocyte-Macrophage Colony Stimulating Factor (GM-CSF). We observed a significant attenuation in the levels of all 12 cytokines within the lungs of mice infected with UM6 compared to lungs from mice infected with UM1 (Fig. 5d). In summary, we propose that high levels of biofilm production attenuate the cytokine response which may explain the increased virulence of *B. pseudomallei* UM6.

### A similar gene expression profile is observed in other *B. pseudomallei* high and low biofilm producing strains

**Fig. 6:**
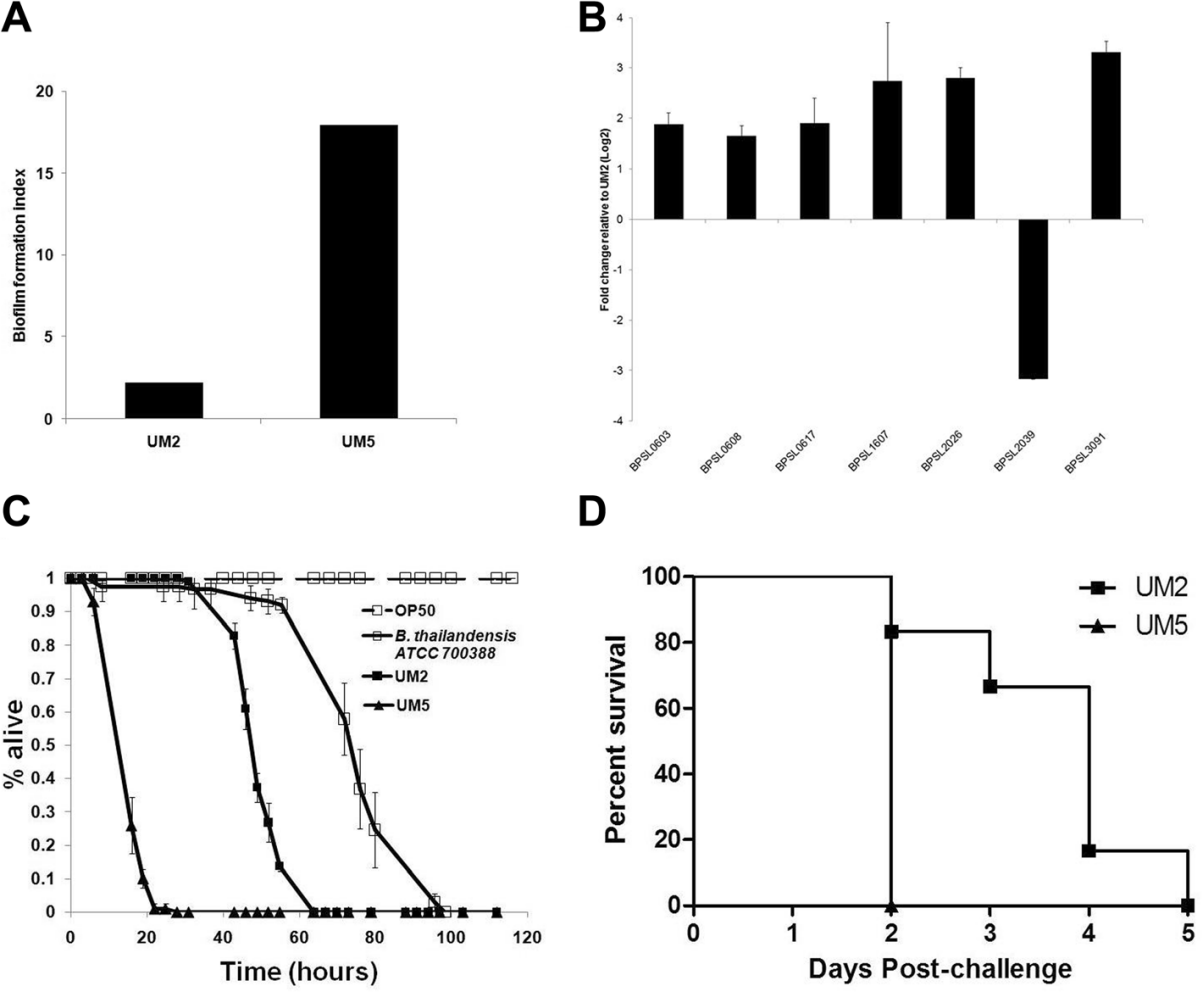
The *B. pseudomallei* high biofilm producer, UM5, also results in faster killing kinetics and over-expression of biofilm-associated genes compared to a second low biofilm producing isolate, UM2. **a** Biofilm index of UM2 and UM5. **b** qRT-PCR analysis of *B. pseudomallei* genes from seven functional categories differentially expressed by RNA-Seq analysis. The results are from a representative of three reproducible independent experiments. **c** Killing assay of *C. elegans* infected with UM2 (black line, closed square), UM5 (black line, closed triangle) and *B. thailandensis* (black line, open square). The graph shows the mean ± SD of three replicates (30 worms/replicate) from a representative of two independent experiments. **d** Mice (n = 5) were challenged intraperitonealy with a lethal dose of *B. pseudomallei* UM5 (triangle) or UM2 (square) and their survival was monitored. Mice challenged with UM5 succumbed to disease significantly faster (within 24 h) than those challenged with UM2 [Logrank (Mantel-Cox) test, *p*-value = 0.0084]

The transcriptional data presented here have identified genes that most likely contribute towards the biofilm phenotype in *B. pseudomallei*. To confirm that the observed gene expression pattern is not restricted to UM6 and UM1, we selected a second high biofilm producing strain (UM5) as well as a low *B. pseudomallei* biofilm producing strain, UM2 (Fig. 6a) to analyse the expression profile. Seven of 11 genes that were modulated in UM6 (Fig. 2b) were analysed, including genes associated with the denitrification pathway, cell envelope and EPS production. Six of the selected genes were also over-expressed in UM5 compared to UM2 (Fig. 6b). Both isolates were also analysed in *C. elegans* (Fig. 6c) and mice (Fig. 6d) infection assays and we observed that the high biofilm *B. pseudomallei* strain UM5 contributed to higher killing kinetics in both animal models.

## Discussion

*B. pseudomallei* biofilm formation may contribute to intracellular survival, dormancy and antibiotic resistance [35] but the mechanism by which this occurs in humans is yet to be demonstrated [8]. To date, only a handful of *B. pseudomallei* biofilm-associated genes have been studied and have mainly focused on single-gene phenotypes. In this study, we demonstrate that biofilm production is a complex process that involves the differential expression of several genes. RNA sequencing analysis performed on low and high biofilm-producing *B. pseudomallei* strains identified genes that contribute to biofilm formation. We identified 563 differentially expressed genes during the formation and growth of biofilm, accounting for about 9.5 % of the total *B. pseudomallei* gene content. The transciptome analysis of biofilm related genes was performed on mid-log bacterial cultures, the pre-biofilm state, to conform to standard sequencing protocols. Keeping in mind that our analysis may not necessarily reflect a true biofilm environment, we subsequently analysed a subset of genes from 7 representative functional groups on UM1 and UM6 cells grown to the stationary phase (mature biofilm state). qRT-PCR analysis demonstrated comparable magnitudes and patterns of gene expression between RNA samples extracted from cells at different growth phases (data not shown). Thus, our findings offer new insights into the different transcriptional landscapes observed between clinical *B. pseudomallei* high and low biofilm producing isolates.

**Fig. 7:**
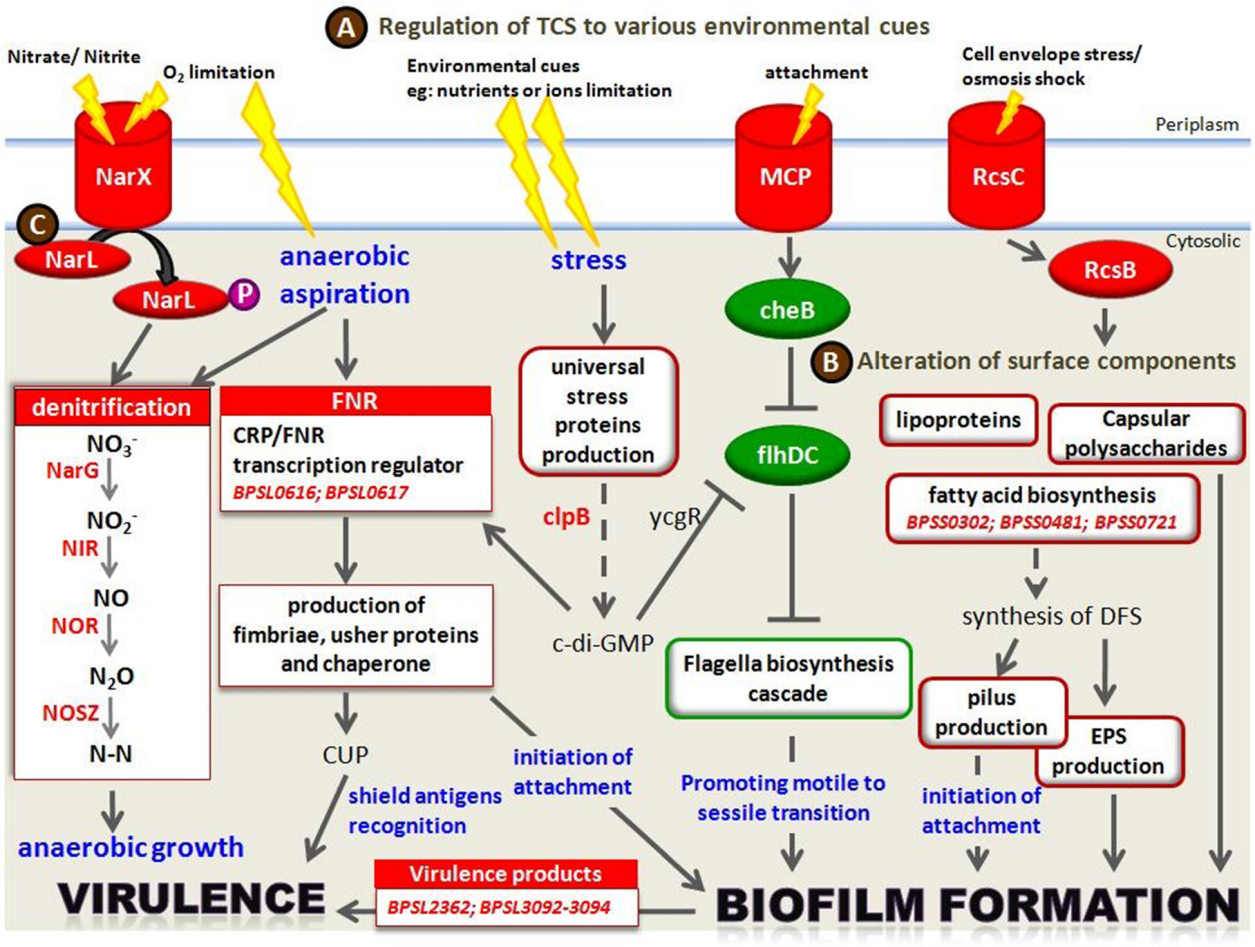
Summary of *B. pseudomallei* genes that contribute to biofilm development. **a** In response to varied environmental signals, *B. pseudomallei* regulates the two-component signal (TCS) transduction system to adapt to environmental changes, by (**b**) transcribing genes that encode proteins involved in the alteration of surface components and components crucial to initiate attachment of planktonic bacterial cells to solid surfaces for microcolony formation. As the biofilm grows, an oxygen-limited environment forms. **c** The high biofilm producing *B. pseudomallei* strain utilises nitrate for anaerobic growth through the denitrification enzyme pathway. Arrow represents activator connection; solid lines represent direct activation; dashed lines represent indirect activation. The boxes and fonts highlighted in red depict the up-regulated biological processes and genes, respectively, while the box highlighted in green depicts the down-regulated biological processes

Biofilm producing pathogens sense environmental signals via the TCS transduction system and adapt to these changes by transcribing genes that planktonic organisms do not [21, 36]. Based on the expression profile of the high biofilm producer, we hypothesise that *B. pseudomallei* also responds to varied environmental signals, for instance pH, temperature, osmotic pressure and oxygen concentration via activation of various TCS (Fig. 7). Upon encountering the environmental cues that stress the cell membranes, *B. pseudomallei* most likely activates the RcsB-RcsC TCS and subsequently regulates genes that encode proteins involved in the alteration of surface components, including capsular polysaccharides, cell envelop biogenesis, lipoproteins, phospolipases and fatty acid biosynthesis that are pivotal for survival of *B. pseudomallei* within the host (Figs. 2a and 7). RcsC sensor kinase is required for biofilm formation in *E. coli* and regulates genes encoding for proteins that are either localised to the envelope or have activities that affect the structure/properties of the bacterial surface [21, 36]. In addition, the cell-to-cell communication small fatty acid signal molecule, diffusible signal factor (DSF), regulates the expression of factors contributing to virulence, antibiotic tolerance and biofilm formation [37, 38]. DSF is synthesized by putative enoyl-CoA hydratase and putative acyl-coA ligase in *Xanthomonas campestris* and *Burkholderia cecocepacia* [39–41]. Three fatty acid biosynthesis genes that encode for Co-A hydratase and ligase are up-regulated in UM6. This, in turn, most likely regulates EPS-associated genes, the core component for maintenance of biofilm architecture and pilus biogenesis-related genes to initiate the attachment of planktonic cells for microcolony formation.

During the growth of micro colonies, methyl- accepting chemotaxis protein MCP (*BPSL2367*), a sensor protein in TCS, is up-regulated in UM6 and subsequently suppresses the regulation of CheB which is responsible for flagella switch (Fig. 7). Down regulation of the flagella biosynthesis cascade seen in the high biofilm producer (Figs. 2a and 3) suggests sessile transition of motile *B. pseudomallei* for biofilm development [28]. Bacterial biofilm formation is known to affect colony morphotype (mucoid vs non-mucoid) [20] and bacterial attachment [42]. This is also true for *B. pseudomallei* biofilm, whereby the low biofilm producer UM1 that exhibits a mucoid colony morphotype, has lower expression levels of pilus, fimbriae, lipoprotein, polysaccharide and EPS-related genes when compared to the wrinkled colony morphotype observed in UM6 (Fig. 1). This observation is similar to a *P. aeruginosa* mutant with a wrinkled colony phenotype capable of forming pronounced pellicles that exhibited increased production of exopolysaccharide and EPS [20].

Recently, the CRP/FNR superfamily protein Bcam1349 of *Burkholderia cenocepacia* (Bcc) was reported as a cyclic diguanosine monophosphate (c-di-GMP) effector in regulating biofilm formation and is involved in virulence [43]. This protein regulates the increased production of cellulose and fimbriae [43]. Two hypothetical proteins (BPSL0616 and BPSL0617) with the CRP/FNR superfamily conserved domain were significantly up-regulated in UM6 (Fig. 2a and Additional file 4). Protein sequence analysis revealed that BPSL0617 has 69 % identity to Bcam1349, suggesting that it is the ortholog of the Bcc Bcam1349 (Additional file 7). Of note, the neighboring genes of these two hypothetical proteins were also up- regulated and a number of these genes are involved in surface protein modification (Additional files 4 and 8). Another member of the CRP/FNR family protein, the c-di-GMP response regulator ClpB heat-shock protein (*BPSL1484*) is also up-regulated in UM6. Furthermore, a cohort of fimbriae related genes, including three chaperone-usher gene clusters (*BPSL1799–BPSL1801*; *BPSL2026–BPSL2028*; *BPSS0091–BPSS0093*) which make up the chaperone-usher fimbriae pathway (CUP) were also over expressed in UM6 (Additional files 4 and 7). Up-regulation of fimbriae genes in biofilm formation seen in this study are similar to that in *Klebsiella pneumoniae* [44] and *E. coli* [45] which promotes adhesion to abiotic surfaces. In addition, *P. aeruginosa* fimbrial CupE/Csu proteins that contain the SCPU sub-domain are reportedly involved in structuring of biofilm [46]. Concomitantly, three hypothetical proteins with the conserved SCPU domain (*BPSL1009*, *BPSL2030* and *BPSL2031*) up-regulated in the high biofilm producer may likely play a role in *B. pseudomallei* biofilm formation.

As the micro colonies mature into a progressively thick biofilm, a nutrient and oxygen-limited environment forms [21, 25] and the anaerobic fitness of the pathogen is pivotal for survival in the biofilm [47]. *P. aeruginosa* uses nitrate as an alternate electron acceptor through a denitrification enzyme pathway during the anaerobic growth of biofilm [48, 49] and this is regulated by ANR, an ortholog of the *E. coli* FNR [48, 49]. This transcriptional profiling suggests that the high *B. pseudomallei* biofilm producer may sense oxygen limitation through the NarX/NarL TCS and subsequently activate the nitrate reductase operon (*BPSL2307*–*BPSL2314*) and several types of nitrate reductases. This suggests that the facultative anaerobe *B. pseudomallei* is also likely to utilise nitrate for anaerobic growth in biofilm through the denitrification pathway regulated by BPSL0617 (Fig. 7). To our knowledge, this is the first report that describes the involvement of the TCS and denitrification enzyme pathways within the anaerobic environment in *B. pseudomallei* biofilm development.

Biofilm formation in other pathogenic bacteria such as *Staphylococcus aureus* and *Streptococcus pneumoniae* has been reported to be associated with altered host immune responses [50, 51]. Our preliminary study suggests that *B. pseudomallei* biofilm is probably a contributing factor towards virulence in both *C. elegans* and BALB/c mice models (Fig. 5a and b). Furthermore, UM6, the high biofilm producer strain failed to elicit the expected cytokine response even though the number of recoverable CFU was similar for the two strains (Fig. 5d). Many inflammatory cytokines referred to as the “core host immune response” molecules commonly seen in general inflammation infections including melioidosis [52], were not over expressed in UM6 infected-mice. This attenuation of *in vivo* inflammation suggests that intracellular *B. pseudomallei* are camouflaged from the host immune defense response by the biofilm causing the host to succumb to the infection. Although our findings challenge the previous report by Taiweechaisupapong et al. [9], both studies are limited by the small number (n = 2) of isolates to conclude a positive or negative association between *B. pseudomallei* biofilm formation and virulence and the analysis of a larger *B. pseudomallei* strain cohort should be undertaken.

## Conclusions

In summary, this is the first report of the complete transcriptome profile of a *B. pseudomallei* biofilm producer. We have identified genes that are likely involved in the development of the *B. pseudomallei* biofilm phenotype, including quorum sensing, motility and surface composition-related genes (Fig. 2a). Interestingly, many of these genes are clustered together in the genome and may be regulated as an operon (Additional file 8). We postulate that the ability to sense various environmental cues and adapt to anaerobic conditions via the denitrification enzyme pathway is pivotal for the formation of *B. pseudomallei* biofilm in the infected host which subsequently allows for persistent infection in chronic melioidosis. *B. pseudomallei* is particularly recalcitrant to antibiotic treatment and this is most likely attributable to biofilm formation. Thus, novel strategies designed to thwart *B. pseudomallei* biofilm formation or to block a specific biofilm developmental stage, such as the use of anti-adhesion agents and inhibitors which interfere with signal transduction, are exciting avenues for the development of potent and bioavailable treatment strategies.

## Methods

### Bacteria

The four clinical *B. pseudomallei* isolates (UM1, UM2, UM5 and UM6) as well as two reference strains, *B. thailandensis* ATCC 700388 [53], and *B. pseudomallei* K96243 [54] used in this study are listed in Additional file 9.

### Sample cultivation, RNA isolation and sequencing

Overnight cultures of *B. pseudomallei* (K96243, UM1 and UM6) were diluted 1:100 in 50 mL BHI broth and were grown at 37 °C until mid- logarithmic phase (OD_600_ = 0.5). Total RNA was isolated from two biological replicates of *B. pseudomallei* UM6 and UM1 harvested at mid- logarithmic growth phase using TRIzol (Invitrogen Life Technologies, CA, USA) according to the manufacturer’s instructions. Residual DNA was completely removed using QIAGEN’s RNase-Free DNase Set and complete DNA removal was validated by performing PCR with the *B. pseudomallei recA* gene primers. The integrity of the total RNA was assessed on the Agilent 2100 Bioanalyzer. Total RNA (10 μg) was subjected to 23 s and 16 s ribosomal RNA removal using the MicrobExpress kit (Ambion, CA, USA). Ribosomal depleted RNA was resuspended in 5 μL elution buffer (Qiagen, GmbH, Germany). A total of 15.5 μL Elute Prime Fragment Mix from the (non-stranded) TruSeq RNA Sample Prep kit (Illumina, CA, USA) was mixed with 4 μL of ribosomal depleted RNA and used for RNA fragmentation followed by cDNA synthesis, end-repair, TruSeq indexed-adapter ligation and PCR enrichment as per the TruSeq RNA sample preparation protocol (Illumina, CA, USA). A total of 6 libraries (2 biological replicates of each bacterial sample), each labelled with a unique index, were multiplexed in one flow cell lane and the sequencing run was performed on the Illumina HiSeq2000 sequencing platform.

### Mapping and analysis of Illumina reads

Sequence reads from each sample were quality pre-processed using the FASTX-toolkit fastq_quality_filter. Trimming was based on the minimum accepted lllumina quality value of 20 and minimum accepted read size of 30 bp. The pre-processed reads were separated between paired and orphan reads using the Python script. Only the paired reads were used in the analysis while orphan reads were discarded. After pre-processing, an average of 7.5 million reads, corresponding to 95 % of the total reads, were mapped to chromosomes 1 and 2 of the *B. pseudomallei* strain K96243 genome sequence (GenBank Accession numbers NC006350 and NC006351). Due to the absence of genome sequences for both UM1 and UM6, this approach may be biased against the accessory genome of *B. pseudomallei*, however, transcripts that mapped to the core genes were the main interest of this study. Mapping generating four total transcriptome profiles (Additional file 2) using the alignment tool TopHat version 2.02 [55] integrated with Bowtie version 0.12.7 [56]. The TopHat default settings were used: 20 alignments per read were allowed with up to 1 mismatch per alignment. To determine differential expression of known transcripts, the resulting aligned reads were analysed by Cuffdiff, a part of the Cufflinks package version 2.02 [55] and expression of those transcripts was reported as fragments per kilobase of transcript per million mapped reads (FPKM). Overall, ≥ 87 % of the generated transcriptome reads were mapped to the *B. pseudomallei* K96243 reference genome. Transcripts with a q-value of ≤ 0.05 and log_2_ fold-change above 1 were considered as differentially expressed transcripts. Sequence reads were deposited in the database of the European Nucleotide Archive with accession number PRJEB6085 and are accessible via http://www.ebi.ac.uk/ena/data/view/PRJEB6085. The sample accession numbers are ERR475457 (UM1;1^st^ replicate), ERR475458 (UM1; 2^nd^ replicate), ERR475459 (UM6;1^st^ replicate) and ERR475460 (UM6; 2^nd^ replicate).

### Hierarchical clustering

Selected data were organized by a hierarchical clustering with the web-based software Cluster 3.0. The clustering algorithm is based on an uncentered correlation metric, with average linkage clustering and visualized using Java Treeview V1.1.3. [52]

### PSORT

The cellular localization of each differentially expressed gene was predicted using PSORTb version 3.0.2 (http://www.psort.org/psortb/). For the run, the following parameters were used: Organism type: Bacteria; Gram stain: Negative. BPSLt38 was excluded from the analysis as it is a tRNA.

### Gene ontology

Functional classifications were carried out based on Comprehensive Microbial Resources (CMR) annotations (www.cmr.jcvi.org) as previously described by Chieng et al. [57].

### Quantitative real-time PCR (qRT-PCR)

qRT-PCRs were performed with total DNase-treated RNA on the Bio-RadiCycler (BioRad Laboratories, USA) to quantify the expression of eleven genes from seven functional categories. Briefly, 20 μL reactions were performed using the iScript™ One-Step RT-PCR kit with SYBR Green according to the manufacturer’s instructions (BioRad Laboratories, USA), primers at a final concentration of 1 μM and a data acquisition temperature of 76 °C. In order to control for variation in RNA concentration, cycle threshold (Ct) values were normalized to *B. pseudomallei* 16 s rRNA that does not change with infection [58]. Primer sets used in this study are shown in Additional file 10.

### Scanning electron microscopy analysis of biofilm formation

Bacteria were cultured as previously described [34]. Briefly, overnight cultures of *B. pseudomallei* (UM1 and UM6) were diluted 1:100 into 50 mL of fresh BHI broth and grown overnight in a shaking incubator at 37 °C. At the end of the incubation, the bacterial density was adjusted to OD_600_ = 1 using a spectrophotometer. For each isolate, 2 mL of bacterial suspension was added to a 12-well plate with 10 mm × 10 mm glass slides placed inside each well. Biofilms were allowed to form on the slides at 37 °C for 48 h following which, the samples were fixed in 4 % (v/v) glutaraldehyde in 0.05 M phosphate buffer (pH 7.0) at 4 °C for 12 h. Subsequently, the samples were washed three times in phosphate buffer, dehydrated through a graded ethanol series, dried in a critical-point drying apparatus with liquid carbon dioxide, sputter coated with gold and viewed using a LEO 1450VP (Electron Microscopy Unit, Universiti Kebangsaan Malaysia).

### Motility assays

Motility assays on solid agar were performed using *B. pseudomallei* that had been cultured on Ashdown’s agar at 37 °C in air for 48 h. Swim agar plates were composed of 1 % tryptone, 0.5 % NaCl, 0.3 % agar whilst 0.5 % agar plates were used to observe swarming. Bacterial cells from an isolated colony was point inoculated into the centre of a swim plate or on the surface of a swarm plate using a sterile toothpick. Plates were incubated at 37 °C in air for up to 72 h, after which the widest colony diameter was measured represented by the circular turbid zone (swim plates) or migratory growth pattern (swarm plates) formed by the bacterial cells migrating away from the point of inoculation [59].

### *C. elegans* survival assays

The wild type *C. elegans* N2 strain used in this study was obtained from the Tan Laboratory at Stanford University. The nematode was propagated on nematode growth medium (NGM) and fed on the normal food source, *E. coli* OP50 [60], at 16 °C.

*C. elegans* survival assays were performed as previously described [61, 62] with minor modifications. *B. pseudomallei* isolates (UM1, UM2, UM5 and UM6), *B. thailandensis* ATCC 700388 and *E. coli* OP50 were grown overnight in 1 mL Brain Heart Infusion (BHI) broth or LB broth at 37 °C. Ten μL of an overnight culture was spread over a small area on 3.5-cm NGM plates and incubated at 37 °C for 24 h. Plates were then allowed to equilibrate to room temperature for 12–24 h before use. Glp worms were prepared as previously described [63] and thirty age-matched Glp worms were transferred to NGM plates seeded with individual *Burkholderia* isolates and incubated at 25 °C. The number of live and dead worms was scored at 4–6 h intervals. For all the assays, *E. coli* OP50 was used in place of *B. pseudomallei* as the negative control.

### Ethics statement

All animal experiments were performed in accordance with the Universiti Kebangsaan Malaysia animal ethics guideline formulated in accordance to the guidelines of the National Health and Medical Research Council of Australia. The experiments were approved by the Universiti Kebangsaan Malaysia Animal Ethics Committee (UKMAEC) under approval number FST/SBB/2010/SHEILA/24-AUGUST/320.

### Mice survival assay

Female BALB/c mice, aged 8–10 weeks old, were obtained from the Animal House Facility, Universiti Kebangsaan Malaysia (UKM). Mice were maintained under specific-pathogen-free conditions in a positive pressure environment at 20–25 °C, subjected to a 12 h light/dark cycle and fed with a protein-enriched diet and water ad libitum. *B. pseudomallei* isolates, UM1, UM2, UM5 and UM6 were cultured as described previously. Mice were challenged intraperitoneally with ~1 × 10^6^ CFU of *B. pseudomallei* UM1 or UM6 and their survival was monitored. The lung, liver and spleen were aseptically removed from mice that succumbed to disease and individually homogenized in 5 mL of PBS. Organ homogenates were serially diluted with PBS and the dilution was plated on Ashdown agar. The bacterial load in each organ was determined as CFU per organ. The remaining homogenates were centrifuged and supernatants were used for cytokine analysis. Statistical analysis on the difference in organ bacterial load was performed using the Mann–Whitney test within the GraphPad Prism version 4.0 (GraphPad Software) software package.

### Measurement of proinflammatory cytokine levels

Mouse cytokine Multi-Analyte ELISArray Kit (Qiagen) was used to measure levels of IL-1α, IL-1β, IL-2, IL-4, IL-6, IL-10, IL-12, IL-17A, Interferon-γ (IFN-γ), Tumour necrosis factor-α (TNF-α), Granulocyte-Colony Stimulating Factor (G-CSF) and Granulocyte-Macrophage Colony Stimulating Factor (GM-CSF) in the organ homogenate supernatants from mice infected with *B. pseudomallei* strains UM1 or UM6. The arrays were performed according to the manufacturer’s instructions. The absorbance was measured at 450 nm with an automated Sunrise ELISA reader (Tecan, Switzerland).

## Additional files

Additional file 1:
**Biofilm production by clinical isolates of** ***B. pseudomallei*** **.** Diagram shows the relative comparison of biofilm formation by 87 *B. pseudomallei* clinical isolates (black bars). *B. thailandensis* ATCC 700388 was used as the reference for calculation of biofilm-forming capacity (red bar). Biofilm formation of *P. aeruginosa* ATCC 27852 (blue bar) and three other *B. pseudomallei* reference strains (K96243, ATCC 23343 and NCTC 13178) (yellow bars) were also included. Additional file 2:
**Analysis of transcriptome sequencing reads mapped to the K96243 genome.**
Additional file 3:
**Summary of significant differentially expressed genes.** Classification of (A) biological function and (B) predicted cellular localization as analysed by PSORT. Pie charts indicate the percentage of up- and down-regulated genes that were significantly regulated in UM6 (high biofilm producer) compared to UM1 (low biofilm producer). Genes were divided into functional categories based on Comprehensive Microbial Resources (CMR) annotations. Additional file 4:
**Transcriptional changes of genes involved in** ***B. pseudomallei*** **biofilm formation.** Shown is the expression profile for significantly regulated genes of UM6 (high biofilm producer) compared to UM1 (low biofilm producer). The genes transcripts are expressed as fragments per kilobase of transcript per million mapped reads (FPKM). Additional file 5:
**qRT-PCR analysis of genes found to be differentially regulated by RNASeq.**
Additional file 6:
**Two putative** ***B. pseudomallei*** **sensor kinases, BPSL2025 and BPSL1634 are members of RcsC.** The amino sequences of two putative *B. pseudomallei* sensor kinases, BPSL2026 (YP_108622.1) and BPSL1634 (YP_108248.1) were aligned with *E. coli* sensor protein RcsC (NP_416722.2) using ClustalW. Conserved residues are highlighted in red and the conserved domains are underlined. Additional file 7:
***B. pseudomallei*** **hypothetical protein BPSL0617 is a member of the CRP/FNR family protein.** The amino sequence of BPSL0617 (YP_107246.1) was aligned with BCAM1349 (YP_002233964.1) using ClustalW. Conserved residues are highlighted in red. Additional file 8:
***B. pseudomallei*** **genes clusters that contribute to biofilm development. Genomic organization of the** ***B. pseudomallei*** **gene clusters that contribute to biofilm development as identified in this study.** Arrows indicate the direction of transcription and colours depict the expression profile. Up and down-regulated genes are coloured in red and green, respectively. Additional file 9:
**Details and biofilm formation index of** ***B. pseudomallei*** **clinical isolates and reference bacterial strains used in this study.**
Additional file 10:
**Primer sequences used for quantitative RT-PCR.**
