# Supplementary figures and images for: Global transcriptional analysis of Burkholderia pseudomallei high and low biofilm producers reveals insights into biofilm production and virulence
Source: BMC Genomics. 2015 Jun 20;16(1):471. doi: 10.1186/s12864-015-1692-0 (PMC4474458; doi:10.1186/s12864-015-1692-0)

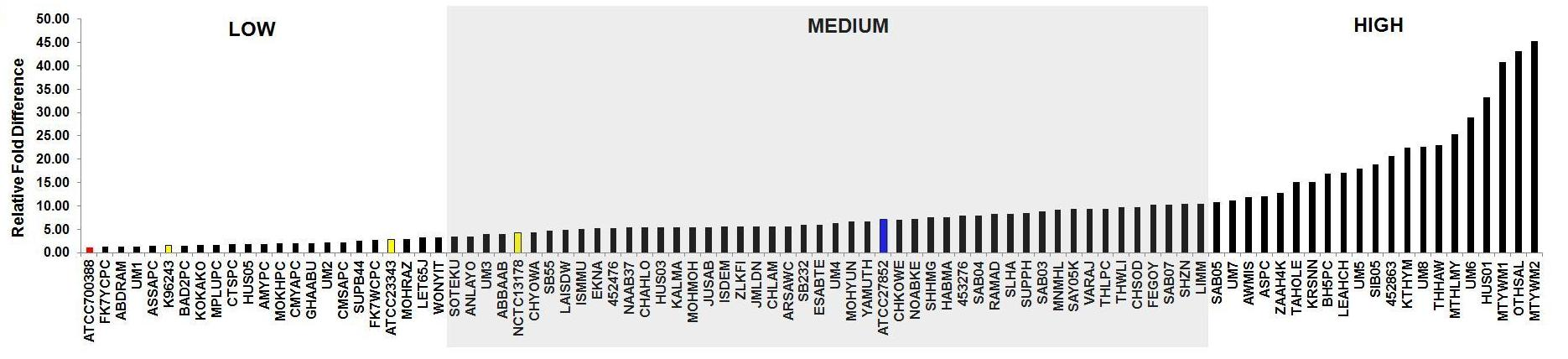

Supplement: Additional file 1: — Biofilm production by clinical isolates of B. pseudomallei . Diagram shows the relative comparison of biofilm formation by 87 B. pseudomallei clinical isolates (black bars). B. thailandensis ATCC 700388 was used as the reference for calculation of biofilm-forming capacity (red bar). Biofilm formation of P. aeruginosa ATCC 27852 (blue bar) and three other B. pseudomallei reference strains (K96243, ATCC 23343 and NCTC 13178) (yellow bars) were also included. [file 12864_2015_1692_MOESM1_ESM.tiff]

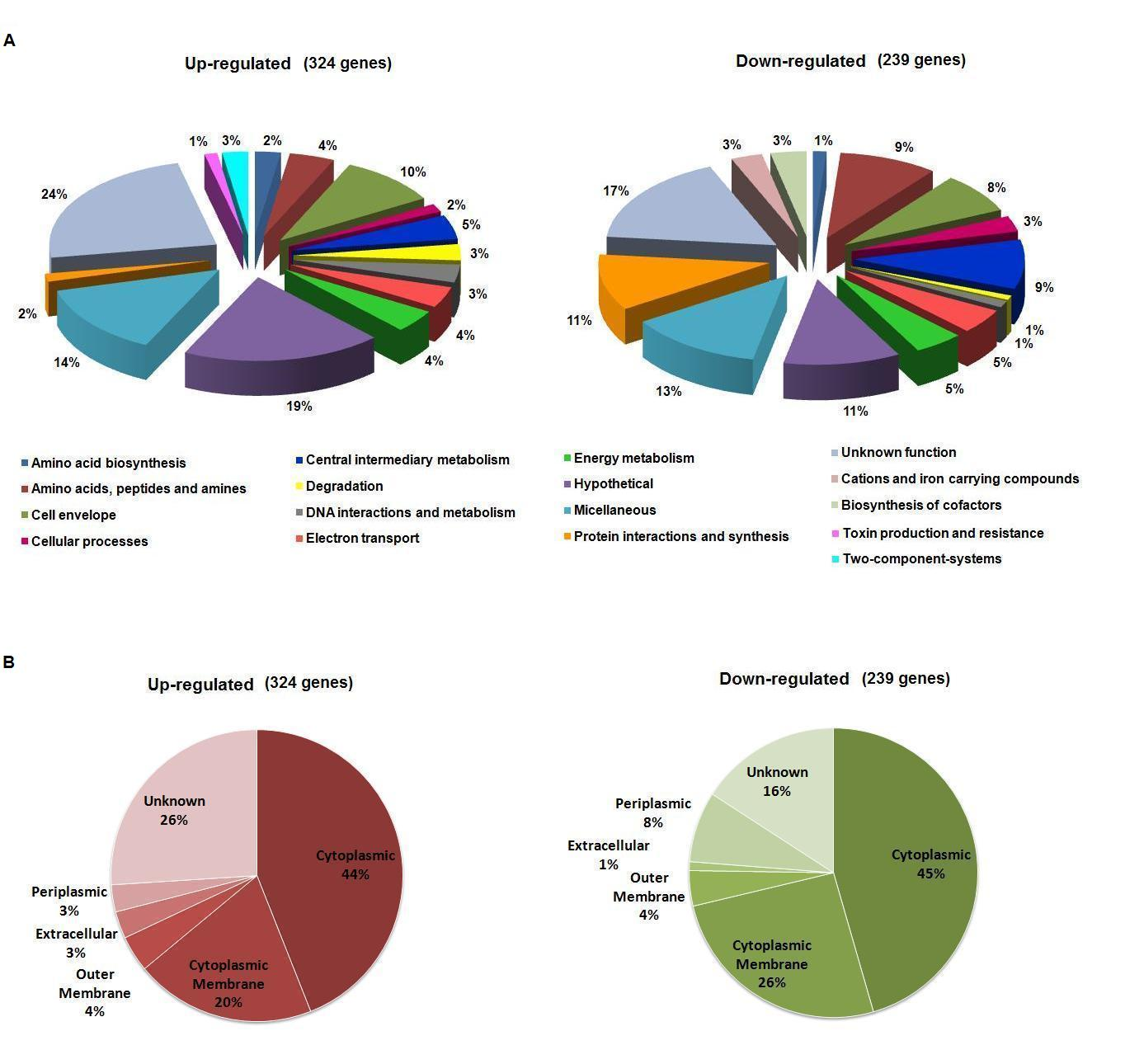

Supplement: Additional file 3: — Summary of significant differentially expressed genes. Classification of (A) biological function and (B) predicted cellular localization as analysed by PSORT. Pie charts indicate the percentage of up- and down-regulated genes that were significantly regulated in UM6 (high biofilm producer) compared to UM1 (low biofilm producer). Genes were divided into functional categories based on Comprehensive Microbial Resources (CMR) annotations. [file 12864_2015_1692_MOESM3_ESM.tiff]

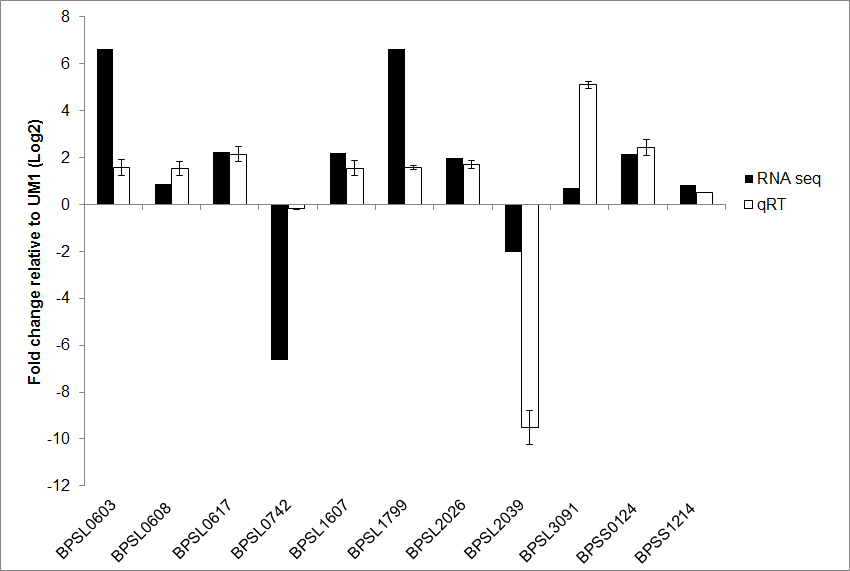

Supplement: Additional file 5: — qRT-PCR analysis of genes found to be differentially regulated by RNASeq. [file 12864_2015_1692_MOESM5_ESM.tiff]

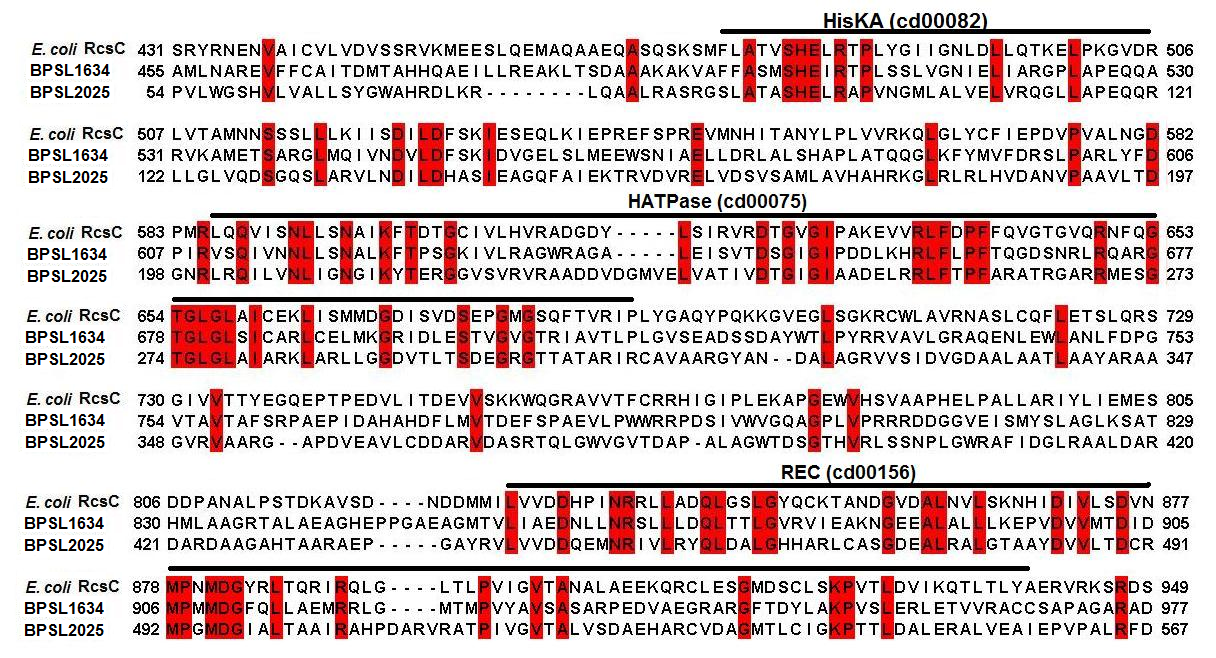

Supplement: Additional file 6: — Two putative B. pseudomallei sensor kinases, BPSL2025 and BPSL1634 are members of RcsC. The amino sequences of two putative B. pseudomallei sensor kinases, BPSL2026 (YP_108622.1) and BPSL1634 (YP_108248.1) were aligned with E. coli sensor protein RcsC (NP_416722.2) using ClustalW. Conserved residues are highlighted in red and the conserved domains are underlined. [file 12864_2015_1692_MOESM6_ESM.tiff]

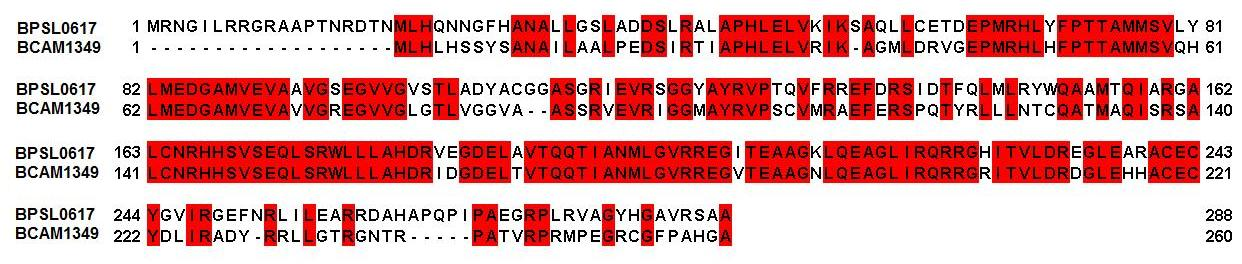

Supplement: Additional file 7: — B. pseudomallei hypothetical protein BPSL0617 is a member of the CRP/FNR family protein. The amino sequence of BPSL0617 (YP_107246.1) was aligned with BCAM1349 (YP_002233964.1) using ClustalW. Conserved residues are highlighted in red. [file 12864_2015_1692_MOESM7_ESM.tiff]

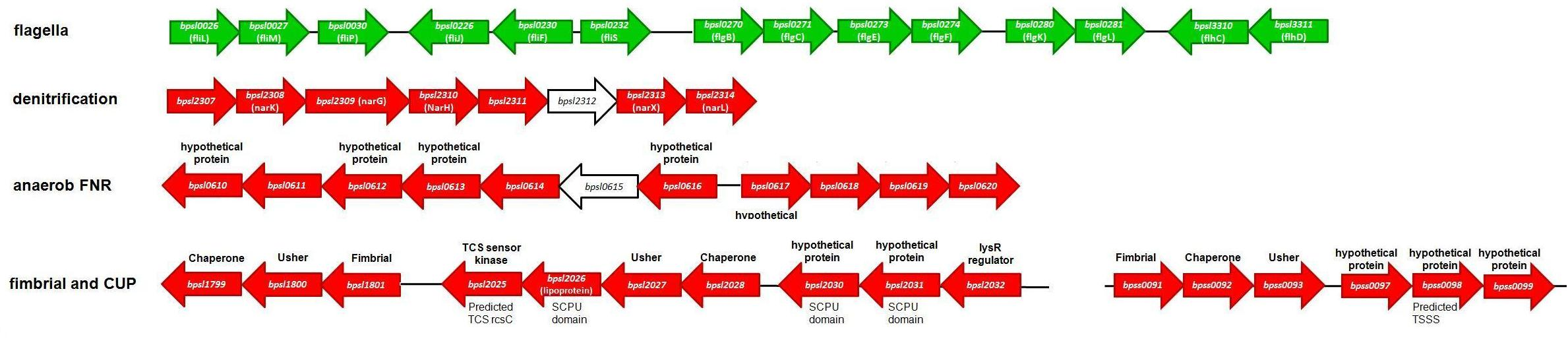

Supplement: Additional file 8: — B. pseudomallei genes clusters that contribute to biofilm development. Genomic organization of the B. pseudomallei gene clusters that contribute to biofilm development as identified in this study. Arrows indicate the direction of transcription and colours depict the expression profile. Up and down-regulated genes are coloured in red and green, respectively. [file 12864_2015_1692_MOESM8_ESM.tiff]
