# Supplementary material for: Global transcriptional analysis of Burkholderia pseudomallei high and low biofilm producers reveals insights into biofilm production and virulence
Source: BMC Genomics. 2015 Jun 20;16(1):471. doi: 10.1186/s12864-015-1692-0 (PMC4474458; doi:10.1186/s12864-015-1692-0)
Supplement: Additional file 2: — Analysis of transcriptome sequencing reads mapped to the K96243 genome. [file 12864_2015_1692_MOESM2_ESM.doc]

**Additional File 2. Analysis of transcriptome sequencing reads mapped to the K96243 genome.**

| **Sample** | **UM1**  **1^st^ replicate** | **UM1 2^nd^  replicate** | **UM6**  **1^st^ replicate** | **UM6 2^nd^  replicate** |
| --- | --- | --- | --- | --- |
| Total cleaned reads | 7,574,314 | 7,633,902 | 7,644,472 | 7,665,200 |
| Total mapped reads | 6,631,315 | 6,676,613 | 6,932,328 | 6,674,775 |
| Percentage of total reads mapped to K96243 (%) | 87.6 | 87.5 | 90.7 | 87.1 |
| Reads mapped to CDS | 5,132 | 5,034 | 4,957 | 5,207 |
| Percentage of CDS mapped (%) | 87.5 | 85.9 | 84.5 | 88.8 |
