# Supplementary material for: Global transcriptional analysis of Burkholderia pseudomallei high and low biofilm producers reveals insights into biofilm production and virulence
Source: BMC Genomics. 2015 Jun 20;16(1):471. doi: 10.1186/s12864-015-1692-0 (PMC4474458; doi:10.1186/s12864-015-1692-0)
Supplement: Additional file 10: — Primer sequences used for quantitative RT-PCR. [file 12864_2015_1692_MOESM10_ESM.doc]

Additional File 9. Primer sequence used for quantitative RT-PCR.

| **Gene ID** | **Forward** | **Reverse** |
| --- | --- | --- |
| *BPSL0603* | CGAAGCTGTAGTTCGTGAAA | AGAATTTCCTGCTGCTGATG |
| *BPSL2026* | TGCAGTAATCCCAGTTCCAT | TGCCGATGTATTTCAGAACG |
| *BPSL1607* | CAGAACAATGGGTTTCACGC | TGAGCGCTCTTGATCTTCAC |
| *BPSL1799* | CCGGTAGAACAGCTTCATCT | TCTATCTGAACATCCGCGAG |
| *BPSL0742* | CTCTTGCTCGAACAGATCAC | CATTCAAACGGTTGTTCGTG |
| *BPSL2040* | ATGGATGGTTCATTTCGTCG | ATGACTCATCCTATGCCGAT |
| *BPSL2039* | AAACATACAGATAGCCGGGA | TCGACAAGAAAAAGGAGCAG |
| *BPSL1607* | ACTACTGCCTGTTCACCTGC | GCCGTTCTCGGTGTTGTACT |
| *BPSL1202* | ATGATCGACGCGTTTGTTGC | CCGGATTGCGGAAGAACCAG |
| *BPSS2016* | CACAACCTGTTCAGCGAAGG | GCCGAGAAACGGAAACTGC |
| *BPSL3091* | AACAAGAAATCGCCATGGTTGG | CCGAAGTTGTAGGCCGACTG |
| *BPSL0603* | CGAAGCTGTAGTTCGTGAAA | AGAATTTCCTGCTGCTGATG |
| *BPSS1103* | ACGATCGCTCGGTATGGTTC | GACAGCGGCAGGAACTTGAA |
| *BPSS0312* | GCGACTTGCAAGAAGAGCCT | GGAGATCTGATGCCGGTACG |
| *BPSS1214* | ATTTCTCGACGACTGTGGGG | TGGCGGAAGTGTTCGTGTTA |
| *BPSL0608* | TCCGAAATCGTTTCCGGCTG | ATAGGAACATTCCGAGCCGC |
| *BPSS0481* | CTGCCGCTACCTGAACGATG | CGCGACTGGTAGAACAGGTA |
| *BPSS0124* | AGCGATGCGACATCCTGATT | GCCTCAAGAACGAAAGCAGC |
| *BPSL0106* | CATGATGATGGGTTTCGGGG | TGCTGATCCATCATGTCGCC |
